# RNA-Binding Proteins at the Host-Pathogen Interface Targeting Viral Regulatory Elements

**DOI:** 10.3390/v13060952

**Published:** 2021-05-21

**Authors:** Azman Embarc-Buh, Rosario Francisco-Velilla, Encarnacion Martinez-Salas

**Affiliations:** Centro de Biología Molecular Severo Ochoa, CSIC-UAM, Nicolás Cabrera 1, 28049 Madrid, Spain; azmane@cbm.csic.es (A.E.-B.); rfrancisco@cbm.csic.es (R.F.-V.)

**Keywords:** RNA-binding proteins, RNA viruses, translation control, stress granules, trafficking factors, IRES elements, ER-Golgi, RNA methylation

## Abstract

Viral RNAs contain the information needed to synthesize their own proteins, to replicate, and to spread to susceptible cells. However, due to their reduced coding capacity RNA viruses rely on host cells to complete their multiplication cycle. This is largely achieved by the concerted action of regulatory structural elements on viral RNAs and a subset of host proteins, whose dedicated function across all stages of the infection steps is critical to complete the viral cycle. Importantly, not only the RNA sequence but also the RNA architecture imposed by the presence of specific structural domains mediates the interaction with host RNA-binding proteins (RBPs), ultimately affecting virus multiplication and spreading. In marked difference with other biological systems, the genome of positive strand RNA viruses is also the mRNA. Here we focus on distinct types of positive strand RNA viruses that differ in the regulatory elements used to promote translation of the viral RNA, as well as in the mechanisms used to evade the series of events connected to antiviral response, including translation shutoff induced in infected cells, assembly of stress granules, and trafficking stress.

## 1. Introduction

**Figure 1:**
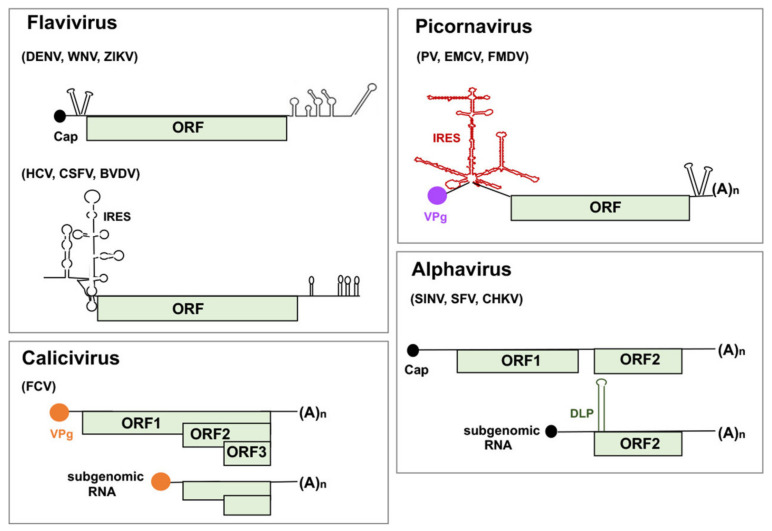
Diagram of the genomic RNA of flavivirus, picornavirus, alphavirus, and calicivirus. The regulatory elements targeted by host RNA-binding proteins (RBPs) described in this review are depicted. The RNA length of the genome is approximately drawn to scale (flavivirus 10–11 kb, picornavirus 7.5–9 kb, alphavirus 12 kb, calicivirus 7.4–8.3 kb). In all cases the open reading frame (ORF) encodes a polyprotein, which is processed into the mature viral proteins. Stem-loops on the 5′ and 3′UTRs of the viral RNA, as well as the downstream stem-loop (DLP) of alphavirus, are schematically represented by hairpins. A black circle depicts the m^7^GTP residue at the 5′ end of the mRNA in flavivirus (Dengue virus (DENV), West Nile virus (WNV), Zika Virus (ZIKV)) and alphavirus (Sindbis virus (SINV), Semliki forest virus (SFV), Chikungunya (CHKV)); the violet and orange circles depict the viral protein (VPg) covalently linked to the 5′ end in picornavirus (Poliovirus (PV), encephalomyocarditis virus (EMCV), foot-and-mouth disease virus (FMDV)) and calicivirus (FCV) RNA, respectively, while (A)n denotes the poly(A) tail at the 3′ end of the RNA in picornavirus, alphavirus, and calicivirus. The internal ribosome entry site (IRES) element present in a subset of flaviviruses (hepatitis C virus (HCV), classical swine fever virus (CSFV), bovine viral diarrhea virus (BVDV)) is depicted by black line. A red line depicts the IRES element of picornavirus.

RNA viruses are a major threat to human health [1]. Their rapid evolutionary capacity favors spread among different organisms, and also can increase the probability of interspecies barriers crossing [2]. Viruses possessing single-stranded positive-strand RNA genomes include a large variety of human, animal, and plant pathogens. Among the different types of viruses belonging to this group, this review will be focused on the RNA regulatory regions present in flavivirus, picornavirus, alphavirus, and calicivirus (Figure 1). Representative members of these genera have been selected taking into consideration the presence of distinct type of regulatory elements within their genomes that are targeted by host RNA-binding proteins (RBPs) performing critical functions on the viral replication cycle. 

The genomic RNA of flaviviruses, exemplified by Dengue virus (DENV), West Nile virus (WNV), or Zika Virus (ZIKV), encodes a single open reading frame (ORF) flanked by untranslated regions (UTR) at each end [3]. The 5′end contains a 7-methylguanosine (m^7^GTP) residue (designated cap), while the long 3′UTR folds into a complex secondary structure that includes several stem-loops critical for viral RNA replication. However, other members of the *Flaviviridae* family differ in the type of regulatory elements controlling their translation and replication, as exemplified by hepatitis C virus (HCV), classical swine fever virus (CSFV), and bovine viral diarrhea virus (BVDV). The genomes of these viruses harbor a functional internal ribosome entry site (IRES) element on their 5′UTR, responsible for the cap-independent translation initiation of the viral RNA [4,5].

Members of the *Picornaviridae* family (such as enterovirus, cardiovirus, and aphthovirus) are characterized by the presence of a long uncapped 5′UTR that contains a covalently linked viral protein (VPg) at the 5′ end (Figure 1). In addition, the RNA genome of all known members of this family is characterized by the presence of an IRES element at the 5′UTR [6], and a poly(A) tail at the 3′ end. The IRES element is located far from the 5′ end, upstream of a single ORF encoding a polyprotein, which is cotranslationally and post-translationally processed into the mature viral proteins [7]. A short 3′UTR folds into a stem-loop structure relevant for viral RNA translation and replication [8]. 

The genomic RNA of alphavirus is capped at the 5′ end and polyadenylated at the 3′ end, as illustrated by Sindbis virus (SINV) and Semliki forest virus (SFV) [9]. The viral genome encodes two separated ORFs (ORF1 and ORF2, encoding nonstructural and structural proteins, respectively). Additionally, a capped subgenomic RNA produced during infection contains a stable downstream-loop (DLP) within ORF2, responsible for recruiting the ribosome at the functional initiation codon [10].

The calicivirus genome also contain a covalently linked VPg protein at the end of an extraordinarily short 5′UTR (8–9 nt) [11], and a polyadenylated 3′UTR [12]. The genomic RNA encodes 2, 3, or 4 ORFs in different frames, and a subgenomic RNA (Figure 1). In contrast to picornavirus RNAs, representative members of this group (feline calicivirus (FCV) or human norovirus) take advantage of VPg to recruit the translation machinery. In all cases, the regulatory elements of the viral RNAs mentioned above are targeted by host factors that contribute either positively or negatively to the viral replication cycle. 

**Figure 2:**
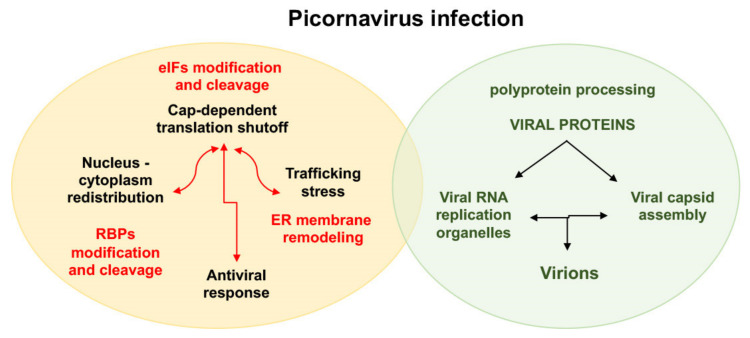
Schematic representation of main events in picornavirus infected cells. Following attachment of the virus to the receptor on the cell surface, internalization, and uncoating leads to release of the viral RNA into the cytoplasm of the host cell. The virus life cycle then proceeds to translation of the polyprotein precursor encoded in the viral genome through interaction with the host translation machinery (green oval). Processing of the polyprotein by viral encoded proteases renders the mature viral proteins required for virus multiplication. Viral RNA replication takes place in viral RNA replication organelles within reorganized cell membranes. Following assembly of viral capsids, the viral RNA is packaged into virions. Viral infection also alters host gene expression through activation of signaling pathways, and modification and/or proteolysis of host factors (eukaryotic initiation factors (eIFs) and RBPs) by viral proteases, inducing cap-dependent shutoff, nucleus-cytoplasm redistribution, trafficking stress, and antiviral response (yellow oval).

Viruses are obligate intracellular parasites that rely on the host translation machinery to produce the proteins required for their replication and spread. Contrary to other viruses, the genome of positive strand RNA viruses is both the viral genome and the mRNA, imposing a close connection between translation and replication events. Early during infection positive-strand RNA viruses activate various signaling pathways and induce cellular membranes modifications rewiring lipid metabolism, leading to the formation of viral RNA replication organelles [13,14]. These entities are the sites where the RNA-dependent RNA polymerase in concerted action with non-structural viral proteins catalyzes the synthesis of intermediate negative strand followed by positive strand RNAs (Figure 2). Following assembly of the structural proteins into viral capsids, the viral RNA genome is packaged into virions. Maturation and release of virions takes place through different mechanisms, and is out of the scope of this review.

Beyond their own encoded proteins, RNA viruses co-opt host RBPs contributing to the recruitment of viral RNAs for viral protein synthesis and the assembly of the complexes regulating viral RNA synthesis. Besides viral RNA translation and replication, RBPs play key roles in all steps of the viral replication cycle (Figure 2), also affecting the localization and the stability of viral RNAs, which have to evade cellular antiviral pathways inducing RNA degradation [15,16].

Our understanding of the diversity of RBPs engaged in virus multiplication has increased in the last decade facilitated by the advances in genomic and proteomic methodologies. These advances have allowed the development of reliable global approaches to detect the interaction of previously unanticipated proteins with viral RNAs. Most of these global techniques are based on cross-linking immunoprecipitation followed by high-throughput sequencing [17]. More recently, viral cross-linking and solid-phase purification (VIR-CLASP) allowed the identification of host proteins associated to 4-thiouridine-labeled viral RNAs in host cells [18]. Similarly, formaldehyde cross-linking with viral RNAs revealed the association of RBPs localized to the endoplasmic reticulum (ER) [19]. In another study, RNA interactome capture (RNA-IC) followed by quantitative mass spectrometry [20] was applied to identify RBPs associated to SINV RNA. Notably, amongst the repertoire of recently discovered RBPs implicated in virus-host interactions there are proteins performing roles previously unconnected to RNA-driven pathways. For instance, it has been suggested that the cellular transport motors dyneins possess RNA binding activity presumably responsible for viral RNA trafficking, uncoating, and reverse transcription [21]. Likewise, the Sec61 translocon complex (a conserved membrane protein complex) or the oligosaccharyltransferase (OST) complex of the ER have been associated with the lifecycle of flaviviruses [22]. Another example is provided by Exoribonuclease Family Member 3 (ERI3), a putative 3′-5′ RNA exonuclease that localizes to the Golgi compartment in uninfected cells, but it is confined to sites of DENV replication in infected cells. This protein has been shown to be involved in viral RNA synthesis via interactions with dumbbell structures in the 3′UTR [23].

In this review we discuss the role of host RNA-binding proteins targeting viral RNA regulatory elements of representative examples of positive-strand virus. These host factors play key roles in the strategies developed by flavivirus, picornavirus, alphavirus and calicivirus to overcome the inhibition of protein synthesis in infected cells, the role of RBPs in the assembly and evading stress granules, and the involvement of ER trafficking host factors targeting regulatory elements of viral RNAs to ribosome-rich compartments. 

## 2. Viral RNA Translation: Strategies to Overcome the Inhibition of Protein Synthesis in Infected Cells

In response to viral infections, cells sense stress signals leading to gene expression reprogramming, typically characterized by a fast inhibition of global protein synthesis (Figure 2). Indeed, many RNA viruses induce a strong translational shutoff in infected cells [15]. The vast majority of cellular mRNAs are characterized by the presence of the cap structure at the 5′ end. The cap protects mRNA from 5′ -3′ exonucleases and allows binding of the eukaryotic initiation factor (eIF)4F complex, consisting of three members, the cap-binding protein eIF4E, the RNA helicase eIF4A, and the scaffolding protein eIF4G. The initiation factor eIF4G interacts with the poly(A)-binding protein (PABP), eIF4E, eIF4A, and eIF3 [24]. Recognition of the 5′cap by eIF4E is followed by recruitment of the 43S complex, consisting of the initiator methionine tRNA (Met-tRNA_i_) as a ternary complex (TC) with eIF2 and GTP (eIF2-TC), and the small 40S ribosomal subunit assisted by additional eIFs. On the other hand, PABP interaction with eIF4G assisted by PABP binding to poly(A)-tail of mRNAs circularizes the 3′ and 5′ ends of the mRNA. Under normal conditions, the 43S complex scans the 5′untranslated region (UTR) of the mRNA until an initiator start codon (AUG) is located in optimum context. AUG recognition allows joining of 60S ribosomal subunit and eIFs release to assemble a translation elongation competent 80S ribosome. 

The general cap-dependent translation initiation in infected cells can be inhibited either by the disruption of the eIF4F complex formation, or by the inhibition of eIF2-GDP recycling [25]. The concentration of eIF2 in the cell is limiting, such that the availability of TC depends upon eIF2B activity, which catalyzes the exchange of GDP for GTP on eIF2. Mammalian cells encode four eIF2α kinases, the protein kinase R (PKR), the heme-regulated inhibitor (HRI), the general control non-derepressible protein 2 (GCN2), and the PKR-like endoplasmic reticulum kinase (PERK), which are activated in response to heat shock, amino acid deprivation, ER stress or viral infection, preventing eIF2 recycling by eIF2B. For instance, cellular infection with DENV induces ER stress resulting in PERK activation and eIF2α phosphorylation [26], while GCN2 can recognize the genomic RNA of SINV blocking early viral translation [27]. However, the subgenomic mRNA of SINV initiate translation in the presence of P-eIF2α through a DLP located downstream of the AUG initiator codon (Figure 1) that stalls the ribosomes on the initiation site of their mRNAs, bypassing the requirement for a functional eIF2 [10]. 

The use of selective mechanisms for translation initiation appears to be a general strategy developed by RNA viruses to sustain viral protein synthesis during cellular translation shutoff. Interestingly, RNA viruses contain regulatory elements on their genome that allow the recruitment of the translation machinery to the appropriate initiation codon, directing accurate viral protein synthesis. As exemplified by picornavirus, flavivirus, calicivirus, and alphavirus infection, viruses subvert host factors for viral RNA translation using diverse strategies, in most cases evading the interference with the abundant cellular mRNA competitors. VPg proteins linked to the 5′end of calicivirus genomes (such as FCV) recruit ribosomes via eIF3, functionally substituting for a cap structure [28]. On the other hand, the 5′UTR of picornavirus RNA harbors a potent IRES element (Figure 1) that governs the synthesis of viral proteins using a cap-independent mechanism [29,30]. Consequently, IRES elements promote viral protein synthesis when cap-dependent translation is impaired [31,32]. 

Although all IRES elements perform a similar function [33], well characterized viral IRES elements lack overall conserved features, and also differ in the requirement of host factors needed to assemble a competent initiation complex [34,35]. Fully functional IRES elements are also present in the viral genome of HCV, CSFV, and BVDV (Figure 1), belonging to the *Flaviviridae* family [36,37]. In contrast, it has been recently found that cap-dependent translation initiation and cap-independent translation initiation mechanisms alternate in DENV infected cells. Interestingly, the weak activity of DENV IRES-like was enhanced in cells expressing the rhinovirus 2A protease, suggesting that the DENV IRES activity enables viral protein synthesis under conditions that suppress cap-dependent translation initiation [38].

Targeting key host translation factors, ribosomal RNAs, or mRNAs at the onset of infection confer a selective advantage for viral RNAs. Cumulative data have shown that picornaviruses induce a shutoff of the host gene expression through the action of viral proteases that cleave host factors (eIFs and RBPs) required for cap-dependent translation initiation (Figure 2), such as eIF4G, PABP, and eIF5B [39,40,41,42,43]. Both, post-translation modification and cleavage of host factors has been extensively documented in picornavirus infection [44]. Enteroviruses encode proteases that cleave eIF4G [45], while encephalomyocarditis virus (EMCV) suppresses cap-dependent translation by activating the translational repressor eIF4E-binding protein 1 (4EBP1) [46]. Cleavage of eIF4G factor by the foot-and-mouth disease virus (FMDV) Leader (L) protease, and rhinovirus 2A protease results in the separation of the PABP and eIF4E binding domains at the N-terminus of the eIF4G protein, preventing cap-dependent translation [42]. Meanwhile, the C-terminal polypeptide of eIF4G carrying the eIF4A and mitogen activated protein kinase-interacting kinase 1 (mnk1)-binding moieties directs IRES-driven protein synthesis [47,48]. 

As already mentioned, IRES activity is resistant to the cleavage of eIF4G [49], which however, causes the shutoff of cap-dependent protein synthesis. Various IRES-binding factors (ITAFs) are targets of picornavirus proteases, as exemplified by the polypyrimidine tract-binding protein (PTB) [50], the far-upstream element binding protein 2 (FUBP2) [51], FUBP1 [52], heterogeneous nuclear ribonucleoproyein K (hnRNPK) [53], serine-arginine rich protein 20 (SrP20) [54], poly C-binding protein 2 (PCBP2) [55], AU-rich element RNA-binding protein (AUF1) [56], or Gemin5 [57], among others. In the case of PTB, the 3C protease of poliovirus (PV) recognizes the three isoforms of this protein generating truncated polypeptides that repress IRES activity. In contrast, cleavage of the repressor FUBP2 in enterovirus 71 (EV71) infected cells results in a fragment that loses its C-terminal region, behaving as an IRES stimulator. Likewise, Gemin5 is cleaved in FMDV infected cells by the action of the Leader (L) protease [57] at similar post-infection times than PABP and PTB cleavage. Proteolysis of Gemin5 renders two detectable C-terminal products, p85 and p57 resulting from two sequential cleavage events. Interestingly, the p85 fragment upregulates IRES activity [58] while the full-length protein behaves as a negative regulator of IRES-dependent translation [59]. Therefore, cleavage of Gemin5 in FMDV infected cells causes a switch in the activity of this protein leading to opposite functions, such that the full-length Gemin5 protein behaves as an antiviral factor, while the p85 fragment appears to act as a proviral factor. Consistent with this hypothesis, a C-terminal fragment of about 21 kDa, released from a sequential cleavage on p85, which contains the IRES repressor activity [58] could be detected in cells expressing the Leader protease but not in FMDV infected cells [57], presumably due to protein instability or degradation. This scenario is reminiscent of eIF4GI proteolysis, where cleavage of the full-length protein in PV infected cells abrogates its cap-dependent translation function, although it stimulates IRES-dependent activity [60].

Gemin5 is a member of the survival of motor neurons (SMN) complex [61]. Additionally, this protein is involved regulation of mRNA translation, and gene expression reprogramming [62]. From the N-terminus to the C-terminus, Gemin5 contains a tryptophan-aspartic (WD) repeat domain [63], a tetratricopeptide (TPR)-like domain responsible for the dimerization of the protein [64], and a non-canonical RNA-binding site [65]. Interestingly, the presence of an intrinsically unstructured region (IDR) within the non-canonical RNA-binding domain of Gemin5 [58] suggested the existence of multiple interactors. This observation prompted the search of cellular RNAs interacting with this singular domain and disclosed its decisive its role in selective translation [66]. In agreement with the existence of multiple interactors for Gemin5, this protein is known to be a cap-binding protein [67], a ribosome interacting protein [68], and a regulator of translation [69,70]. Gemin5 also interacts with two genetically distant viral IRES elements (present in FMDV and HCV genomes), downregulating IRES-dependent translation [59]. In addition, a recent report reported the redistribution and enhanced RNA-binding affinity of Gemin5 to SINV replication areas in infected cells [20], and also in the interactome of Severe Acute Respiratory Syndrome-Coronavirus-2 (SARS-CoV-2) [71] (BioRxiv preprint), suggesting a general role in viral infection. Relocation of RBPs to viral factories is indicative of the pivotal role of host factors for viral multiplication, and suggests a concerted action of RBPs for the assembly of viral replication complexes.

## 3. Dual Role of RBPs in the Assembly and Evading Stress Granules 

**Figure 3:**
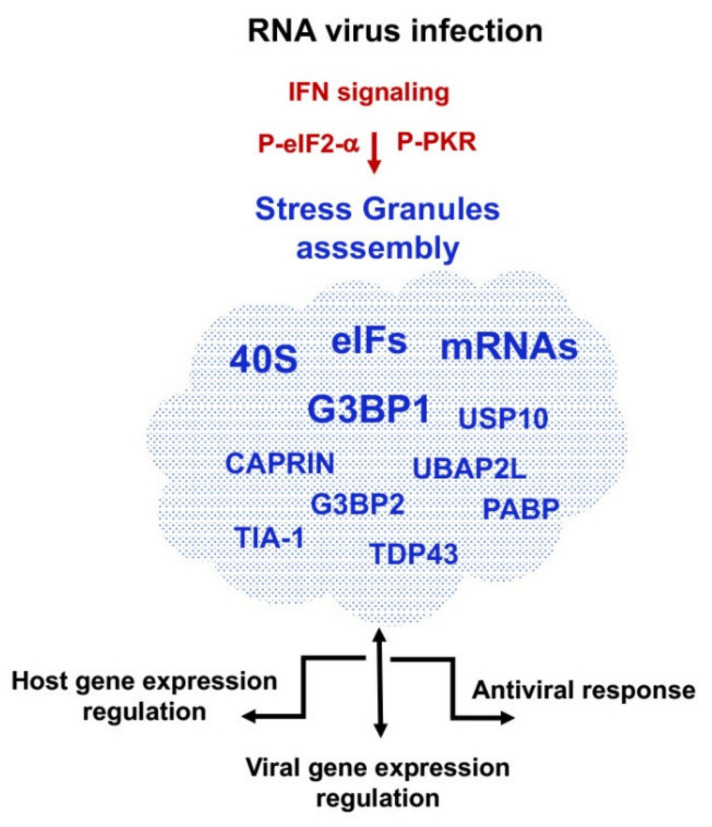
Pathways leading to the assembly of stress granules (SGs) in RNA virus infected cells and outcome in gene expression regulation. Early infection events trigger the interferon (IFN) signaling, concomitant to protein kinase R (PKR) activation via dsRNA and eIF2α phosphorylation, leading to SGs assembly, generating a cascade of pathways that impact on host gene expression, viral gene regulation, and antiviral response. Blue letters denote main components of SGs.

Strong stresses such as those induced by viral infections trigger a rapid inhibition of protein synthesis, ultimately leading to polysome disassembly and formation of stress granules (SGs) [72]. SGs are cytoplasmic membrane-less dynamic aggregates containing stalled pre-initiation complexes, which are thought to serve as sites of mRNA storage during the cell stress response [73]. Generally, SGs gather ribonucleoprotein particles (RNPs), enriched in mRNAs, eIFs, 40S ribosomal subunits, and RBPs. Marker proteins of SGs are Ras-GAP SH3 domain binding protein (G3BP1-2), Caprin, Ubiquitin Associated Protein 2 Like (UBAP2L), Ubiquitin Specific Peptidase 10 (USp10), PABP, Fragile X Mental Retardation Protein (FMR1), TAR DNA-binding protein 43 (TDP43), T-cell-restricted intracellular antigen-1 (Tia1), and Tia-1 related protein (TiaR) [73,74] (Figure 3).

One of the pathways leading to SGs assembly is connected to phosphorylation of the α subunit of eIF2 (eIF2α). During viral infections the eIF2α kinase PKR is activated by dsRNA, promoting aggregation of stalled mRNPs [75]. High levels of P-eIF2α hampers GDP/GTP exchange in the ternary complex, and thus, inefficient delivery of the initiator Met-tRNA_i_ [25]. Similarly, inactivation of translation initiation induced by mTOR inactivation, leads to low levels of eIF4E-BP phosphorylation, thereby sequestering eIF4E and causing a reduction of cap-dependent translation initiation. Yet, viruses can undergo replication under cap-dependent inhibition avoiding both the host stress response and the antiviral response [72].

Targeting of RNPs into SGs is carried out by multiple RNA-protein, protein-protein, and RNA-RNA interactions [76]. Recent reports have shown that SGs are characterized by the presence of proteins carrying intrinsically disordered regions (IDR). The unfolded region of IDR proteins not only confers multitasking properties [77], but also reduces the concentration that allows phase-separation, a feature shared by several SGs markers. In addition, the capacity of RNA to entail both RNA-RNA and RNA-protein interactions modulates the properties of IDRs. In line with this view, G3BP1 has been reported as a molecular switch that controls RNA-dependent liquid phase separation [78]. 

G3BP exists in two forms, G3BP1 and G3BP2. G3BP1 has been reported to provide the core for most SGs interactions [79]. This protein belongs to a family of RBPs that link tyrosin/kinase receptors-mediated signaling and RNA metabolism [80]. It is a widely conserved multifunctional protein that comprises at the C-terminal region two RNA-binding motifs, the RNA recognition (RRM) and the arginine-glycine rich (RGG) motifs [81]. G3BP1 interacts with 40S ribosomal subunits through the RGG motif, which is also required for G3BP-mediated SGs formation [74]. Among the factors interacting with G3BP1 that presumably contribute to the dynamism of SGs, Caprin facilitates aggregation of G3BP1 with RNA while USp10 abrogates SGs assembly [74]. Likewise, Tia1, contains three N-terminal RRMs, an IDR, and a prion like related domain, promotes SGs assembly [82]. A recent study of the Caprin interactome in stressed cells revealed the interaction with numerous RBPs that also contain IDRs, including Gemin5 [83]. Consistent with previous reports, the interactors of Caprin1 under stress conditions were primarily annotated to the ribosome, spliceosome, and RNA transport pathways. 

RNA viruses have developed several strategies to overcome the antiviral response due to SGs, manipulating the G3BP interactome and using different mechanisms of action across the viral infection cycle. For instance, G3BP1 functions in viral VPg-dependent translation initiation, contributing to the assembly of translation complexes on the calicivirus RNA genome by facilitating ribosome recruitment [84]. On the contrary, G3BP1 interacts with the FMDV IRES element, negatively controlling translation [85]. However, in cells infected by enteroviruses, G3BP1 is redistributed to replication complexes disrupting canonical stress granules, hence facilitating viral replication [86,87]. Similarly, alphavirus, including SFV and chikungunya virus (CHIKV) recruit G3BP into viral replication complexes. This interaction, which takes place through the viral non-structural protein 3 (nsP3) and the nuclear transport factor 2 (NTF2)-like domain of G3BP, concentrates viral replication complexes and recruits the translation initiation machinery, promoting translation of viral mRNAs [88]. Indeed, alphavirus utilize SGs proteins for the assembly of viral RNA complexes [89], taking advantage of the IDR domains of the viral non-structural protein nsP3.

Although the involvement of G3BP1 in viral gene expression is extensively documented, the mechanisms involved in virus spread appear to be different. Members of the picornavirus family, such as PV, coxsackievirus B3, EMCV, and mengovirus can either block or disassemble SGs [90,91,92]. In particular, during early stages of PV infection, G3BP1 leads to SGs assembly, likely reducing the rate of viral RNA translation and replication. However, this protein is targeted by the viral protease 3C [86] inactivating its role as antiviral factor. In other picornavirus, such as FMDV, the activity of the viral proteases L and 3C induce the cleavage of both G3BP1 and G3BP2 [85,93]. Specifically, G3BP1 is cleaved during FMDV infection yielding two fragments, p55 corresponding to N-terminal domain and an undetected fragment corresponding to the C-terminal region, which harbors RRM and RGG motifs. Thus, cleavage of the G3BP1 protein appears to be a strategy used by picornavirus to counteract the antiviral function of SGs. Furthermore, since G3BP1 is a key component of SGs and also innate immune activation through PKR, viruses that target G3BP1 for degradation can simultaneously inactivate SGs assembly and impair innate immune response. 

In the case of DENV infection, G3BP1 has been reported to display proviral activity [94]. In support of this enhancing effect, the complex G3BP1-G3BP2-Caprin1 (required for the accumulation of IFN stimulated genes (ISGs) and for translation of PKR and interferon induced proteins with tetratricopeptides repeats (IFIT) mRNAs) interacts with the subgenomic sfRNA of DENV inhibiting the antiviral activity of the complex, ultimately facilitating viral replication. G3BP1 also regulates the efficiency of viral replication in HCV infected cells through the interaction with the 5′ end of the minus strand in conjunction with non-structural proteins associated to replication complexes [95]. Consistent with these findings, both G3BP1 and Tia-1 are required for HCV RNA and protein accumulation at early times of infection, and also for the assembly and egress at late infection times. Furthermore, HCV infection triggers PKR phosphorylation, down-regulating synthesis of ISGs [96,97], thereby facilitating viral multiplication. 

Increasing the repertoire of viruses that utilize SGs components for viral multiplication, recent data have shown that coronavirus also induce a shutoff of host gene expression during the first hours after infection [98,99,100,101,102]. The interactome of SARS-CoV-2 revealed multiple interactions between viral and host proteins, including members of translational machinery, proteins involved in SGs formation, and the ER stress response [103]. Specifically, the viral nucleocapsid (N) protein that harbors an IDR, interacts with G3BP1 and G3BP2. Hence, it would not be surprising that other RNA viruses utilize the conformational flexibility of disordered domains of proteins in conjunction with the dynamic structural folding of RNAs for host factor recruitment.

## 4. Vesicular Trafficking Host Factors Targeting Regulatory Elements of Viral RNAs

The involvement of ER-Golgi trafficking factors in the recruitment of viral RNAs to replication organelles has received great interest in the last years [14,104,105]. Vesicular traffic within the early secretory pathway is mediated by coat complex protein I (COPI) and coat complex protein II (COPII) coated vesicles. Transport in the ER-Golgi shuttle is performed by COPII in the anterograde direction, and by COPI in the retrograde direction. However, while COPII-coated vesicles appear to be involved exclusively in the export of secretory proteins and lipids from the ER, COPI-coated vesicles are involved in both anterograde and retrograde transport between the ER-Golgi intermediate compartment and the Golgi, as well as in intra-Golgi transport. Proteins involved in the retrograde transport include the coatomer subunit alpha (COPA), the Golgi Brefeldin A resistant guanine nucleotide exchange factor 1 (GBF1), the small GTPase ADP-ribosylation factor 1 (Arf1), and the intracellular membrane trafficking Ras-related protein 1b (Rab1b), a GBF1 effector molecule [106]. 

The ER membranes are involved in multiple cellular functions, ranging from synthesis, folding, secretion, and degradation of proteins to lipid biogenesis. Viruses also make use of ER functions to promote their life cycles. In particular, replication of enterovirus requires the cellular protein GBF1. Upon activation, Arfs associate with membranes regulating numerous steps of membrane homeostasis. During early stages of enterovirus infection, Arf1 is recruited to the replication organelles, colocalizing with the viral proteins and mature virions. Nonetheless, although Arf3, Arf4, Arf5, and Arf6 were located on the replication organelles, only Arf1 and Arf6 depletion increased the sensitivity of replication to GBF1 inhibition [107]. Other studies have shown that altered expression of Arf4 and Arf5 inhibited DENV virus secretion at an early pre-Golgi step [108], while changes in Arfs expression delayed HCV viral RNA replication [109].

The anterograde transport pathway participates in the life cycle of various RNA viruses, including picornaviruses and flaviviruses [108,110]. Factors involved in the anterograde transport include the secretion associated RAS related GTPase 1a (Sar1a), the guanine nucleotide exchange factors Sec12, Sec23/24, Sec13/31, and Rab1b [111]. In the case of Rab1b, different results have been reported for HCV infection cycle. One study suggested that inhibition of Rab1b function inhibits the release of virus particles [112]. However, another work [109] reported that this protein affects viral RNA replication and translation. Therefore, the mechanism of action of the different trafficking factors in virus infection still remains elusive. 

**Figure 4:**
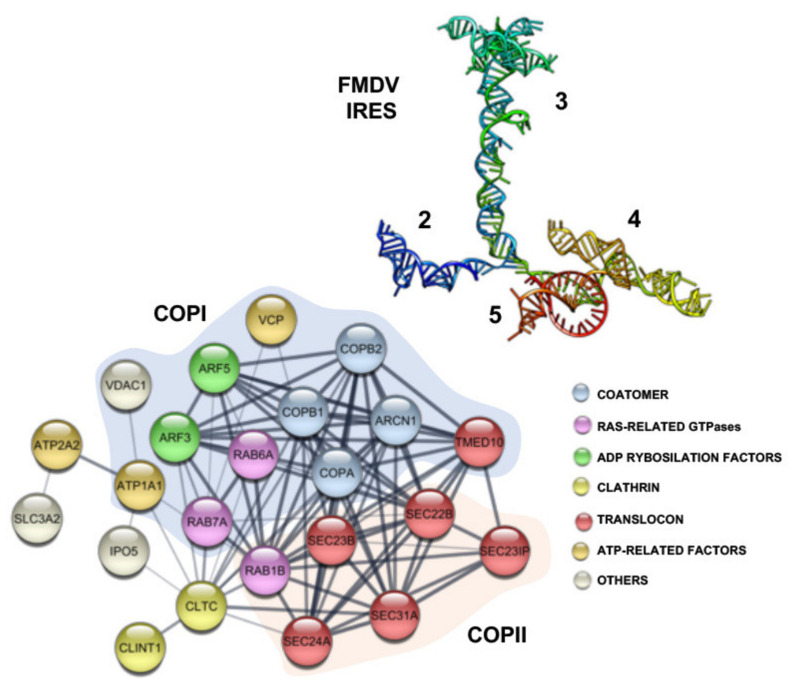
Host factors interacting with domain 3 of the FMDV IRES element. RNA structure model of the IRES element based on RNA Selective 2′ Hydroxyl Acylation analyzed by Primer Extension (SHAPE) probing (33) (top). The central domain (3) is colored in green. The functional association of proteins identified using the tRNA-scaffold methodology (123), interacting with domain 3 of the IRES, related to ER-Golgi vesicles trafficking was generated with STRING software (https://string-db.org). Factors involved in the anterograde (COPII) or retrograde (COPI) transport are marked by orange and blue shadow, respectively. Proteins are represented by circles and colored based on their functional grouping.

The interplay between trafficking factors and viral RNA motifs could be an integral part of the regulation of viral RNA function. Following uncoating, the first intracellular step of picornavirus life cycle requires translation of the viral genome, which is governed by the IRES element. Beyond driving internal initiation of translation, a recent work showing the interaction of the FMDV IRES element with proteins such as Rab1b and Arf5 supported the hypothesis that these proteins contribute to locate IRES-driven RNA at the ER-Golgi in a rich ribosome environment [113]. This hypothesis is consistent with the ER localization of RNAs carrying the EMCV IRES [114,115]. Colocalization of Rab1b on the ER membranes [116] depends upon the GTP status of Rab1b [117,118]. This pathway may occur concomitantly to eIFs and IRES-transacting factors mediated translation [119]. In agreement with this possibility, several members of the anterograde and retrograde transport were identified using a combined RNA-structure affinity and proteomic approach (Figure 4), further supporting the notion that specific members of the ER-Golgi transport pathway mediate recognition of the IRES element.

The interaction of the FMDV IRES element with Rab1b and Arf5 reinforce the importance of exploring novel RNA-protein complexes to understand the intricacies of host-pathogen interface. Rab1b is a key regulatory protein involved in COPI and COPII transport [106], whereas Arf5 is an integral member of the Golgi [120]. In support of the RNA-binding capacity, Arf5 and Rab1b interact with IRES transcripts in vitro in the absence of other factors, although they promote different effects on IRES-dependent translation in living cells. However, this differential effect on viral translation seems to be achieved through different mechanisms of action. Overexpression of a dominant negative mutant of Rab1b induced a decrease of IRES-dependent translation, concomitant to ER-Golgi disruption and impairment of RNA localization, while interaction of the IRES element with Arf5 may sequester the mRNA on the trans-Golgi, likely interfering IRES-driven translation [113].

The regulatory elements of viral RNAs share structural features relevant for their function, such that conserved structural elements associate RBPs connected to its relevance for the viral replication cycle. It is significant considering that in addition to short sequence motifs, RNA recognition by RBPs is profoundly affected by secondary and/or tertiary RNA structure. In spite of the high sequence variability of RNA viruses, the secondary structure of the IRES element is conserved among all FMDV serotypes [121]. Moreover, experimental evidence indicates that the FMDV IRES is organized in structural domains that include tertiary interactions impacting on its cap-independent activity [122] (Figure 4). Thus, the RNA architecture imposed by the presence of specific structural domains mediates IRES function. Towards this respect, implementation of a robust RNA-protein interaction approach allowed detecting a variety of ribonucleoprotein complexes associated with specific structural domains of the FMDV IRES element [123] (Table 1). In support of the validity of this approach, well known IRES-interacting factors, such as Poly(rC)-binding protein 2 (PCBP2) or ErbB3-binding protein 1(Ebp1), were retrieved in this RNA-affinity binding methodology. Furthermore, some of these proteins have been reported to affect positively or negatively IRES-dependent translation (Table 1), as in the case of FUBP2 [51], heterogeneous nuclear ribonucleoprotein A1 (hnRNPA1) [124], or synaptotagmin binding cytoplasmic RNA interacting protein (SYNCRIP) [125]. However, others are involved in multiple steps of RNA-dependent pathways such as mRNA polyadenylation, RNA stability or SGs assembly [126,127,128,129,130,131,132], reflecting the large versatility of many RBPs. 

**Figure 5:**
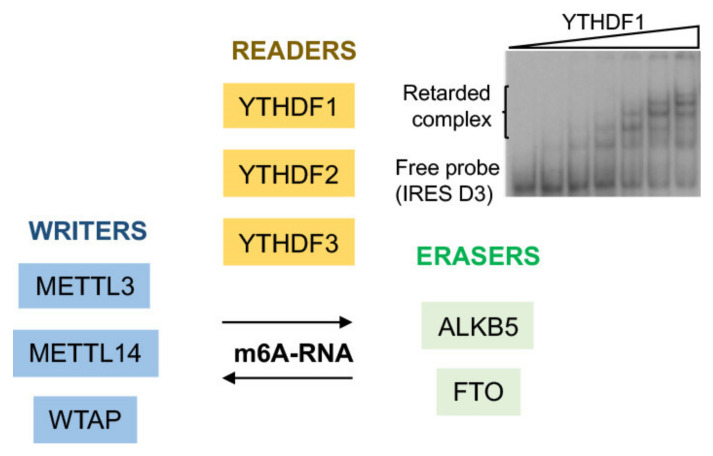
Diagram of m^6^A RNA modification enzymes. The proteins known as writers, readers, and erasers are depicted. The image on the right shows the interaction of YTH N6-methyladenosine RNA binding protein 1 (YTHDF1) protein with labeled domain 3 the FMDV IRES in RNA retarded gel shift assay.

Surprisingly, YTH N6-methyladenosine RNA binding protein 1 (YTHDF1) and YTHDF2 were identified among the novel factors interacting with the IRES element (Table 1) [123]. These proteins are sensors of adenosine methylation (m^6^A) on the RNA [133,134]. Although the potential effect on FMDV viral replication remains to be studied, the intrinsic RNA interaction ability was verified by RNA electrophoretic mobility shift assay (Figure 5). RNA methylation occurs on 0.2% of nucleotides in polyadenylated RNAs, of which m^6^A accounts for 50% of all methylation [135,136]. This process involves various enzymes, designated as writers (m^6^A methyltransferases), erasers (m^6^A demethyltransferases), and readers (effectors that bind to m^6^A) (Figure 5). Adenine methylation on mRNA mostly occurs on the RRACH (R = purine, H = A, C, or U) consensus motif when it is preferentially located on the coding sequence and 3′UTR, near the stop codon region [137]. The methyltransferase complex methyltransferase like (METTL3-METTL14) with the accessory proteins WT1 associated protein (WTAP), KIAA1429, RNA binding motif protein 15 (RBM15), and Zinc finger CCCH domain-containing protein 13 (ZC3H13), methylates adenosines in nuclear RNAs [138]. Demethylation of m^6^A is carried out by alpha-ketoglutarate-dependent dioxygenase (FTO) or Alpha-ketoglutarate-dependent dioxygenase alkB homolog 5 (ALKHB5) [139]. The YT521-B homology (YTH) family regulates the function of m^6^A. Specifically, YTHDF1 enhances ribosome loading on mRNAs via interaction with eIF3A and eIF3B [134]. In contrast, binding of YTHDF2 to mRNAs promotes RNA degradation via relocation to P-bodies [133]. 

Methylation of adenosine on viral mRNAs has multiple effects on virus replication [140,141]. For instance, EV71 infection increases the expression of METTL3, METTL14, YTHDF1–3 and YTHDC1 in the host cells, while the expression of FTO was reduced. In line with this view, knockdown of METTL3 repressed EV71 replication and mutation of m^6^A modification sites of the viral genome resulted in decreased virus replication, suggesting that m^6^A modification might enhance EV71 life cycle [142]. In this case, METTL3 interacts with the viral RNA polymerase enhancing its sumoylation and ubiquitination, boosting viral replication. 

Given that m^6^A has a wide range of effects on mRNA translation, it is reasonable to assume that mechanisms influencing viral protein synthesis could be affected by m^6^A methylation. A recent study reported that HCV IRES-dependent translation is stimulated by m^6^A methylation via YTHDC2 [143], consistent with earlier reports indicating that m^6^A methylation favors cap-independent translation of certain cellular mRNAs [144,145]. However, there are controversial results for other RNA viruses. For instance, other authors have reported that m^6^A modification suppresses HCV viral particle production through the action of YTHDF proteins [146], whereas m^6^A in ZIKV and CHIKV RNA decreases virus replication [140,147]. These results suggest that m^6^A modification could regulate RNA functions in various manners. However, there are open questions for unclear results in RNA viruses that need to be elucidated in future studies. 

## 5. Concluding Remarks

To accomplish the replication cycle, viruses have developed diverse strategies taking advantage of RNA regulatory elements that recruit RBPs and other host factors in all stages of the infection cycle. Most significantly, the regulatory elements of viral RNAs contain structural features relevant for their function, such that conserved structural elements associate RBPs connected to its relevance for the viral replication cycle. Highlighting the plasticity of RNA-protein interactions to achieve a large variety of specific functions, there are many ways to complete the replication cycle. Notably, the translation field has accumulated insights into the regulatory mechanisms occurring in both the host cell and the RNA virus translation during the entire viral replication cycle, and consequently, the antiviral state. 

Host factors are essential at all stages of the virus life cycle. Thus, besides performing critical roles in viral RNA translation and replication, host proteins participate in early infection steps, such as internalization and uncoating [105,148] and in late stages of the infection cycle, such as virion packaging and release [149,150,151,152]. Host RBPs also perform various antiviral functions ranging from the recognition of the viral RNA to the restriction of viral replication. Viral infection induces a fast host response that triggers the production of interferons (IFNs), and subsequently, of ISGs [153]. RNA virus infections activate retinoic acid-inducible gene-I (RIG-I)-like (RLRs) and Toll-like (TLRs) pathogen recognition receptors (PRRS) inducing the antiviral response via transcriptional activation of IFNs, cytokines, and other antiviral proteins such as the Interferon-induced transmembrane proteins (IFTM) [154]. Conversely, viruses have developed a diversity of strategies that counteract the host antiviral responses [43,155], such as the proteolysis of key host proteins involved in antiviral immunity by viral proteases. Representative examples of targets of proteolysis are the RNA-helicases retinoic acid-inducible gene-I [RIG-I), melanoma differentiation associated gene 5 (MDA5) and Laboratory of Genetics and Physiology 2 (LGP2), the innate immune adaptor molecules mitochondrial antiviral signaling (MAVS) and Tol/IL1 receptor domain-containing adaptor (TRIF) inducing interferon-beta proteins, respectively, and the p65-RelA subunit of the nuclear factor kappa B (NFKB) [156,157,158,159,160,161].

In this review we have revisited representative examples of positive strand viral RNA translation initiation, the strategies developed to overcome the inhibition of protein synthesis in infected cells, the role of RBPs in the assembly and evading stress granules, the involvement of ER trafficking host factors targeting regulatory elements of viral RNAs to ribosome-rich compartments, and the influence of viral mRNAs m^6^A methylation in viral replication. The different combinations of RNA-protein complexes executing diverse mechanisms of action suggests that there is much to learn about virus–host interaction interface, highlighting the need to explore the multifaceted role of RNA-protein and protein-protein interactome in infected cells. Ultimately, dissecting the relationship between viral RNAs and host RBPs is expected to expand our understanding of virology and RNA biology in general, and also, to guide the development of novel antiviral approaches. 

## Figures and Tables

**Table 1:** Examples of host RNA-binding proteins interacting with the FMDV IRES.

Protein	Function	Reference
HNRNPC	Splicing, translation	Flynn et al., 2015 [17]
HNRNPD/AUF1	Stability, translation	Cathcart and Semler, 2014 [56]
KHSRP/FUBP2	Stability, translation	Chen et al., 2013 [51]
SYNCRIP/HNRNPQ	Splicing, stability, translation	Kim et al., 2004 [125]
HNRNPUL1	Splicing, transcription	Pacheco et al., 2008 [129]
HNRNPA1/A0	Splicing, stability, translation	Tolbert et al., 2017 [124]
SFPQ	Splicing, translation	Cosker et al., 2016 [132]
NUDT21	mRNA polyadenylation	Brumbaugh et al., 2018 [131]
FAM120A	mRNA transport, stability	Kelly et al., 2019 [127]
IGF2BP3/IMP3	Stability, translation	Jia et al., 2018 [128]
LARP4B	Stability, translation	Mattijssen et al., 2021 [130]
UPF1	NMD, stability	Garcia-Moreno et al., 2017 [20]
CAPRIN1	Stress granules assembly	Anderson and Keersha, 2008 [73]
ATXN2L	Stress granules assembly	Singh et al., 2021 [126]
UBAP2L	Stress granules disasssembly	Anderson and Kedersha, 2016 [75]
YTHDF1	Sensor of m^6^A, translation	Wang et al., 2015 [133]
YTHDF2	Sensor of m^6^A, RNA stability	Wang et al., 2014 [134]
